# The relationship between intraoperative hypothermia and postoperative delirium: The PNDRFAP study

**DOI:** 10.1002/brb3.3512

**Published:** 2024-05-15

**Authors:** Jiahan Wang, Lei Zhu, Chuan Li, Yanan Lin, Bin Wang, Xu Lin, Yanlin Bi

**Affiliations:** ^1^ Department of Anesthesiology Qingdao Municipal Hospital Qingdao China; ^2^ Department of Medical Scientific Research Qingdao Municipal Hospital Qingdao China

**Keywords:** cognitive function, intraoperative hypothermia, mediation analysis, postoperative delirium, risk factor

## Abstract

**Objective:**

Our study aimed to investigate the correlation between intraoperative hypothermia and postoperative delirium (POD) in patients undergoing general anesthesia for gastrointestinal surgery.

**Methods:**

The study comprised 750 participants from the Perioperative Neurocognitive Disorder Risk Factor and Prognosis (PNDRFAP) study database, which ultimately screened 510 individuals in the final analysis. Preoperative cognitive function was evaluated using the Mini‐Mental State Examination (MMSE). The occurrence of POD was determined using the Confusion Assessment Method, and the severity of POD was evaluated using the Memorial Delirium Assessment Scale. Logistic regression was employed to scrutinize the association between intraoperative hypothermia and the incidence of POD, and the sensitivity analysis was conducted by introducing adjusted confounding variables. Decision curves and a nomogram model were utilized to assess the predictive efficacy of intraoperative hypothermia for POD. Mediation analysis involving 10,000 bootstrapped iterations was employed to appraise the suggested mediating effect of numeric rating scale (NRS) scores at 24 and 48 h post‐surgeries. The receiver‐operating characteristic (ROC) was utilized to evaluate the effectiveness of intraoperative hypothermia in predicting POD.

**Results:**

In the PNDRFAP study, the occurrence of POD was notably higher in the intraoperative hypothermia group (62.2%) compared to the intraoperative normal body temperature group (9.8%), with an overall POD incidence of 17.6%. Logistic regression analysis, adjusted for various confounding factors (age [40–90], gender, education, MMSE, smoking history, drinking history, hypertension, diabetes, and the presence of cardiovascular heart disease), demonstrated that intraoperative hypothermia significantly increased the risk of POD (OR = 4.879, 95% CI = 3.020–7.882, *p* < .001). Mediation analyses revealed that the relationship between intraoperative hypothermia and POD was partially mediated by NRS 24 h after surgery, accounting for 14.09% of the association (*p* = .002). The area under the curve of the ROC curve was 0.685, which confirmed that intraoperative hypothermia could predict POD occurrence to a certain extent. Decision curve and nomogram analyses, conducted using the R package, further substantiated the predictive efficacy of intraoperative hypothermia on POD.

**Conclusion:**

Intraoperative hypothermia may increase the risk of POD, and this association may be partially mediated by NRS scores 24 h after surgery.

## Graphical abstract

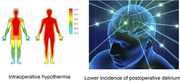

## 1 INTRODUCTION

Postoperative delirium (POD) is a frequent and significant complication affecting the central nervous system in patients following surgery. Typically, it manifests within 1–7 days after the surgical procedure, or before discharge, with a particularly elevated incidence during the first 1–3‐day post‐surgeries (Evered et al., 2018). Delirium is defined as an acute impairment of attention and cognition. Common symptoms include confusion, reduced attention, impaired orientation, reduced cognitive abilities, personality and mood changes, and even delusions and hallucinations (Blazer & van Nieuwenhuizen, 2012). One of the most prominent symptoms is an inability to concentrate and a change in alertness. It is most seen in elderly patients, patients with preexisting neurocognitive impairments, and patients undergoing complex or emergency surgery. Studies have shown that the pathogenesis of POD includes (1) cholinergic theory, (2) abnormal energy metabolism, (3) inflammatory response theory, (4) oxidative stress response theory, (5) exogenous toxins and free radical damage, and (6) ischemic hypoxic brain injury (Zheng et al., 2017). Neurochemical changes and imbalances affect the neurotransmitter system in the brain. The onset of POD not only imposes a heightened financial burden on patients and extends the duration of hospitalization but also often leads to a deterioration in the quality of life, which, in turn, hinders patients from resuming a normal and functional lifestyle (Witlox et al., 2010).

A necessary condition for metabolism and life activities is normal body temperature. Humans maintain a constant body temperature through autonomy and behavioral thermoregulation. Under normal circumstances, the body temperature is constant in the range of 36.0–37.3°C, and when the body temperature is lower than 36°C during surgery, it is called hypothermia (Forbes et al., 2009). According to literature reports, about 50% of surgical patients have a postoperative body temperature lower than 36°C, and 33% have a body temperature lower than 35°C. The behavioral thermoregulation ability is lost during anesthesia surgery. It is not enough to maintain a constant body temperature by simply relying on the thermoregulation center to regulate the body's heat production and heat dissipation. The patient's body temperature can change with the ambient temperature (Sessler, 2000). In addition, intraoperative operations, such as disinfection, infusion, application of anesthetic drugs, and low‐temperature environment in the operating room, will lead to the occurrence of hypothermia in patients, so there is a common phenomenon of body temperature imbalance in the perioperative period (Yi et al., 2015). Perioperative hypothermia may damage the coagulation function and immune function of patients, increase the postoperative infection rate of patients, and lead to cardiovascular function disorders and delayed recovery (Frank et al., 1997; Leslie & Sessler, 2003).

At present, the clinical awareness of the harm of perioperative hypothermia is generally insufficient, and perioperative routine insulation has not been achieved except for major cardiovascular operations. At present, the research on the influence of intraoperative body temperature on cognitive function is still in the exploratory stage, and there are different findings on the relationship between low temperature and cognitive function. In addition, some studies have found that patients with postoperative cognitive function decline have lower intraoperative body temperature. A small observational study reported that in patients undergoing cardiac surgery, those with POD were found to have lower minimum intraoperative temperature (*p* = .035) (Rudiger et al., 2016). But there is still no direct evidence that low body temperature can cause POD occurrence. Due to the similar mechanisms and manifestations of POD and cognitive dysfunction, we made the hypothesis that perioperative hypothermia could be one of the risk factors of POD.

## 2 METHODS

### 2.1 PNDRFAP study

The data for this study were obtained from voluntary participants enrolled in the Perioperative Neurocognitive Disorder Risk Factor and Prognosis (PNDRFAP) study, spanning from January 2020 to May 2021. The PNDRFAP initiative is dedicated to the early diagnosis and prevention of neurocognitive disorders among the Han population in northern China. Initiated in 2019, it constitutes a substantial cohort study designed to analyze the risk factors associated with perioperative neurocognitive impairment.

### 2.2 Participants

This study, registered under Clinical registration number ChiCTR2000033639, obtained approval from the Ethics Committee of Qingdao Municipal Hospital. Participants in the PNDRAFP study were individuals aged between 40 and 90 years, categorized under the American Society of Anesthesiologists (ASA) physical status I–III. Patients need to have complete preoperative cognitive function and no communication impairment, receive adequate education, and complete preoperative mental status exam. Moreover, participants were required to have a Mini‐Mental State Examination (MMSE) score of no less than 24, and they should not have any severe psychological disorders or hearing impairments.

**FIGURE 1:**
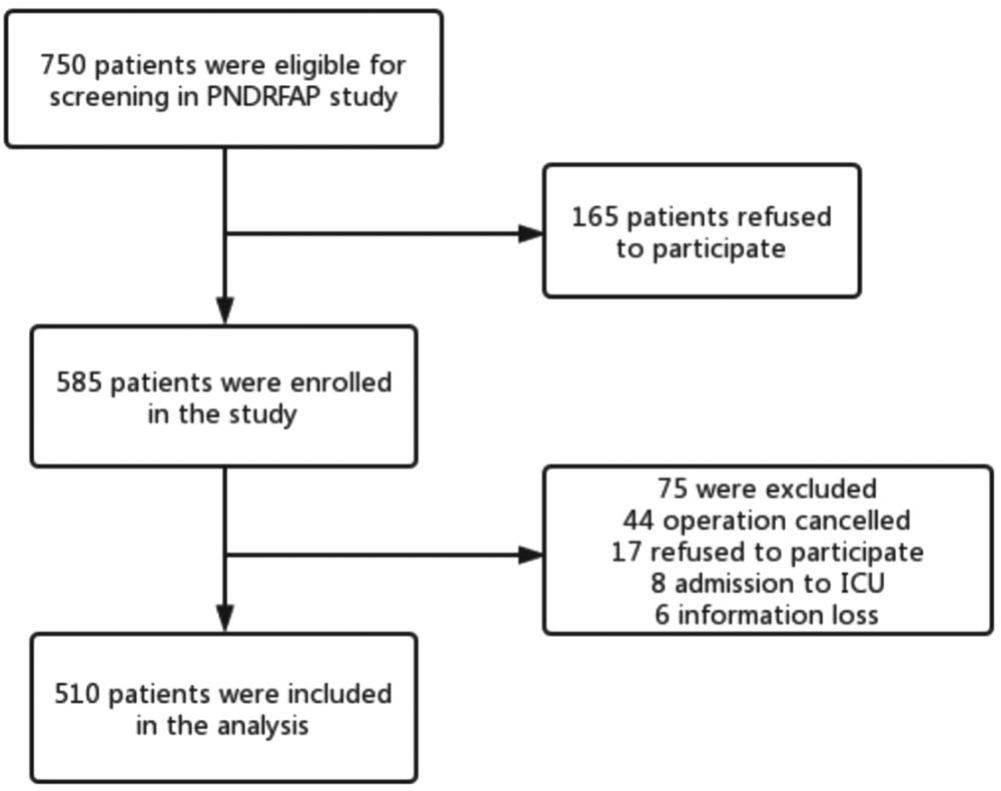
The flow diagram showed that 750 participants were initially screened for the Perioperative Neurocognitive Disorder Risk Factor and Prognosis (PNDRFAP) studies, and finally 510 participants were included in the data analysis.

A preliminary test identified six covariates to be included in the logistic regression analysis. The estimated incidence of POD was 10%, and a 20% loss‐to‐follow‐up rate was assumed. Consequently, a sample size of 750 cases was calculated using the formula (6 × 10 ÷ 0.1 ÷ 0.8 = 750). After screening, 510 participants were included in the PNDRFAP study (Figure 1).

The study involved preoperative interviews with patients, during which baseline data were collected. This information encompassed patient age, gender, body mass index (BMI), ASA physical status, and years of education. Additionally, details regarding any comorbidities and medical history were extracted from the patient's past medical records. The entire process of gathering patient history and conducting physical examinations for this study was carried out by anesthesiologists.

None of the participants received preoperative medication. Before surgery, participants were instructed to abstain from eating for 8 h and from drinking for 6 h. In the operating room, standard monitoring procedures were implemented, including electrocardiogram, pulse oxygen saturation (SpO_2_), noninvasive blood pressure, and nasopharyngeal temperature. Venous access was established, and 3 mL of whole venous blood was collected. General anesthesia was administered to participants in the PNDRFAP study. Anesthesia induction involved the use of sufentanil (0.2–0.5 μg/kg), cisatracurium (0.15–0.2 mg/kg), and etomidate (0.15–0.3 mg/kg). Intraoperative analgesia was maintained through a continuous infusion of remifentanil (0.25–2 μg/kg/min). Cisatracurium was administered every 40 min after induction and discontinued 1 h before the conclusion of the procedure. Depending on the depth of anesthesia, the sevoflurane inhalation rate was adjusted to a range between 0.5% and 3%. The operation time, anesthesia time, intraoperative blood loss, and fluid input were also meticulously documented. If the intraoperative body temperature is lower than 36°C, it is defined as intraoperative hypothermia, and a lower temperature limit alarm is set. Any occurrence of intraoperative hypothermia was carefully observed, and insulation measures would be taken in time.

Following the surgery, the patient was transferred to the anesthesia recovery room, where their condition was closely monitored for a duration of 30 min. If no abnormalities were observed during this period, the patient was then returned to the ward. Postoperatively, intravenous patient‐controlled analgesia (PCA) was employed for pain management. The PCA regimen comprised a combination of butorphanol tartrate injection (10 mg) and dolasetron hydrochloride injection (5 mg), which was diluted to 100 mL with 0.9% sodium chloride solution. The objective was to maintain the patient's numerical pain score below 3 points.

### 2.3 Psychometric measures

To assess a patient's cognitive function before surgery, neurologists utilized the MMSE. Patients with an MMSE score below 24 were excluded from this study.

The Pittsburgh Sleep Quality Index (PSQI) score was applied to test the sleep characteristics of the patients preoperatively. PSQI is a self‐reported questionnaire evaluating sleep quality during the last month, including sleep quality, sleep efficiency (sleep duration in ratio to the total time spent in bed), sleep latency (minutes spent before falling asleep), sleep disturbances, nocturnal sleep duration (hours), daytime dysfunction, and sleep medication use. The total score ranges from 0 to 21, which means that the higher the score, the worse the sleep.

Postoperatively, delirium assessment was conducted twice daily, from 9:00 to 10:00 a.m. and from 2:00 to 3:00 p.m., spanning a duration of 1 to 7 days or until discharge. The responsibility for performing the delirium assessment rested with an anesthesiologist. The Confusion Assessment Method was employed to define POD, which encompasses four clinical criteria: (1) acute onset and fluctuating course; (2) inattention; (3) disorganized thinking; and (4) altered level of consciousness. For a delirium diagnosis, both criteria (1) and (2) had to be met simultaneously, along with either criteria (3) or (4). The severity of POD was gauged using the Memorial Delirium Assessment Scale. Participants were categorized into two groups based on the presence or absence of POD: the POD group and the no POD group.

### 2.4 Statistical analysis

The normality of the measurement data was evaluated using the Kolmogorov–Smirnov test. Data conforming to a normal distribution were reported as mean ± standard deviation, whereas non‐normally distributed data were presented as median (p25, p75) or as a percentage (%). Baseline characteristics between the intraoperative hypothermia group and the intraoperative normal body temperature group were assessed using the chi‐square (*χ* ^2^) test for categorical variables and the Mann–Whitney *U* test for continuous variables.

First, a comparison was made between the perioperative and postoperative characteristics of the two groups.

Second, binary logistic regression analysis was carried out to investigate the association between POD and intraoperative hypothermia. Sensitivity analyses were conducted by (1) including four covariates (age within the range of 40–90, gender, years of education, and MMSE) in the multivariate logistic regression analysis to assess the relationship between POD and intraoperative hypothermia; (2) introducing additional covariates (hypertension, type 2 diabetes, coronary heart disease, smoking, and alcohol intake) in the multivariate logistic regression analysis to explore the relationship between POD and intraoperative hypothermia for patients aged 40–90, gender, years of education, and MMSE.

Third, we employed linear regression models based on Baron and Kenny's methods to scrutinize the mediation effect of numeric rating scale (NRS) scores at 24 and 48 h after surgery on the relationship between intraoperative hypothermia and POD. In the first equation, we regressed the mediator (NRS scores after surgery) on the independent variable (intraoperative hypothermia). In the second equation, we regressed the dependent variable (POD) on the independent variable. In the third equation, we regressed the dependent variable on both the independent variable and the mediator variable. We considered mediation effects to be present if the following criteria were met: (1) Intraoperative hypothermia was significantly associated with NRS scores after surgery; (2) intraoperative hypothermia was significantly associated with POD; (3) NRS scores after surgery were significantly associated with POD; and (4) the association between intraoperative hypothermia and POD was attenuated when NRS scores after surgery (the mediator) were included in the regression model. Furthermore, we determined the significance of the mediation effect using 10,000 bootstrapped iterations.

Decision‐making was employed to characterize the predictive value of intraoperative hypothermia, and nomograms and calibration curves were utilized to visualize and validate the predicted models. The DynNom package in R was utilized to create a Dynamic nomogram (URL: https://thedile.shinyapps.io/dynnomapp‐1/) for predicting the likelihood of POD.

The statistical significance level was set at *p* < .05.

Finally, the efficacy of intraoperative hypothermia in predicting POD was assessed using the area under the curve (AUC) of receiver‐operating characteristic (ROC) curves.

The statistical analysis was carried out using Stata MP16.0 (Solvusoft Corporation, Inc.), GraphPad Prism version 8.0 (GraphPad Software, Inc.), and R software version 4.4.1 (R Foundation for Statistical Computing).

## 3 RESULTS

### 3.1 Participant characteristics

This study initially enrolled a total of 750 participants, with 510 individuals included in the final analysis. The exclusion of 240 participants was based on the criteria outlined in Figure 1. Demographic and clinical characteristics of the included participants are summarized in Table 1. No significant differences were observed in terms of sex, underlying diseases (such as diabetes and coronary heart disease), drinking history, and smoking history between the two groups (*p* > .05). However, the two groups exhibited significant differences in age, years of education, preoperative MMSE score, preoperative PSQI score, history of hypertension, preoperative hemoglobin levels, preoperative albumin levels, ASA grade, and BMI (*p* < .05). The incidence of POD was found to be 17.6% (*n* = 90/510), with 62.2% (*n* = 56/162) occurring in the intraoperative hypothermia group and 9.8% (*n* = 34/348) occurring in the intraoperative normal body temperature group. A statistically significant difference in the occurrence of POD between the two groups was observed (*p* < .05) (Table 3).

**TABLE 1:** Demographic and clinical characteristics of participants.

Variable	Intraoperative hypothermia group (*n* = 162)	Intraoperative normal body temperature group (*n* = 348)	*p* Value
Age, year	68.0 (60.0, 78.0)	64.0 (56.0, 72.0)	.002
Female, *n* (%)	64 (39.5)	143 (41.1)	.734
Education, year	9.0 (5.0, 10.0)	9.0 (9.0, 12.0)	.006
MMSE score, point	26.0 (25.0, 27.0)	27.0 (26.0, 28.0)	<.001
PQSI score, point	10.0 (6.0, 12.0)	8.0 (5.0, 10.0)	<.001
Hypertension, *n* (%)	95 (58.6)	166 (47.7)	.021
Diabetes, *n* (%)	49 (30.2)	86 (24.7)	.187
CHD, *n* (%)	50 (30.9)	86 (24.7)	.144
Smoking history, *n* (%)	63 (38.9)	125 (35.9)	.518
Drinking history, *n* (%)	64 (39.5)	113 (32.5)	.120
Hb, g L^−1^	126.0 (107.3, 140.0)	132.0 (123.3, 144.0)	<.001
Alb, g L^−1^	38.4 (35.2, 40.6)	38.6 (36.6, 40.9)	.049
BMI, kg m^−2^	24.1 (22.0, 26.7)	24.8 (23.0, 27.0)	.010
ASA grade, *n* (%)			.010
I, *n* (%)	6 (3.7)	19 (5.5)	
II, *n* (%)	112 (69.1)	269 (77.3)	
III, *n* (%)	44 (27.2)	60 (17.2)	

*Note*: Continuous variable use Student's *t* test or Mann–Whitney *U*, categorical variable use chi‐square test. The numerical variables of normal distribution are statistically described by average standard deviation. Non‐normally distributed numerical variables are statistically described by interquartile range (IQR). Categorical variables are statistically described by sample size, percent.

Abbreviations: ASA, American Society of Anesthesiologists; BMI, body mass index; CHD, cardiovascular heart disease; Hb, hemoglobin; MMSE, Mini‐Mental State Examination.

### 3.2 Perioperative clinical characteristics of the two groups of patients

From Table 2, significant differences (*p* < .05) persisted between the two groups regarding intraoperative infusion volume, history of blood transfusion, bleeding volume, urine volume, operation time, anesthesia time, and episodes of hypotension among the patients.

**TABLE 2:** Intraoperative factor comparison of participants.

Variable	Intraoperative hypothermia group (*n* = 162)	intraoperative normal body temperature group (*n* = 348)	*p* Value
Infusion volume, mL	1600.0 (1100.0, 2500.0)	1100.0 (600.0, 1200.0)	<.001
History blood transfusion, *n* (%)	32 (19.8)	15 (4.3)	<.001
Bleeding volume, mL	50.0 (20.0, 150.0)	20.0 (5.0, 72.5)	<.001
Urine volume, mL	300.0 (125.0, 600.0)	100.0 (0.0, 300.0)	<.001
Intraoperative hypotension, *n* (%)	100 (61.7)	88 (25.3)	<.001
Operation time, min	170.0 (105.0, 241.3)	92.5 (55.0, 140.0)	<.001
Anesthesia time, min	230.0 (155.0, 315.0)	140.0 (95.0, 190.0)	<.001

*Note*: Continuous variable use Student's *t* test or Mann–Whitney *U*, Categorical variable use chi‐square test. The numerical variables of normal distribution are statistically described by average standard deviation. Non‐normally distributed numerical variables are statistically described by interquartile range (IQR). Categorical variables are statistically described by sample size, percent.

### 3.3 Postoperative features exhibited distinctions between the two groups of patients

Anesthesia complications during PACU, NRS scores 24 h after surgery, NRS scores 48 h after surgery, hospitalization expenses, and POD occurrence showed significant differences between the two groups, as shown in Table 3 (*p* < .05). However, there were no significant disparities noted in postoperative hospital stays (*p* > .05).

**TABLE 3:** Postoperative comparison of participants.

Variable	Intraoperative hypothermia group (*n* = 162)	intraoperative normal body temperature group (*n* = 348)	*p* Value
PACU complication, *n* (%)	21 (13.0)	25 (7.2)	.034
NRS score 24 h after surgery, point	2.0 (1.0, 3.0)	2.0 (1.0, 2.0)	<.001
NRS score 48 h after surgery, point	2.0 (0.0, 2.0)	1.0 (0.0, 2.0)	<.001
Postoperative hospital stay, day	6.0 (5.2, 6.7)	5.7 (5.1, 6.6)	.133
Hospitalization expenses, RMB	44731.9 (31999.3, 73836.4)	28315.5 (17546.9, 44731.9)	<.001
POD occurrence, *n*(%)	56 (34.6)	50 (13.4)	<.001

*Note*: Continuous variable use Student's *t* test or Mann–Whitney *U*, categorical variable use chi‐square test. The numerical variables of normal distribution are statistically described by average standard deviation. Non‐normally distributed numerical variables are statistically described by interquartile range (IQR). Categorical variables are statistically described by sample size, percent.

Abbreviations: POD, postoperative delirium; NRS, numeric rating scale.

### 3.4 The relationship between intraoperative hypothermia and POD

We conducted a comparison of intraoperative hypothermia between patients with POD and those without POD. Binary logistic regression analysis revealed that intraoperative hypothermia (OR = 4.879, 95% CI = 3.020–7.882, *p* < .001) remained highly significant and acted as a risk factor for POD (refer to Table 4). To ensure the robustness of the findings, sensitivity analyses were carried out using two models, each incorporating additional covariates in the studies. In both sensitivity analyses, intraoperative hypothermia remained a stable and significant risk factor for POD (Table 4). These sensitivity analyses confirmed the robustness of our findings.

**TABLE 4:** The logistic regression analysis and sensitivity analysis among intraoperative hypothermia and postoperative delirium (POD).

	Unadjusted		Adjusted1		Adjusted2	
	OR (95%CI)	*p* Value	OR (95%CI)	*p* Value	OR (95%CI)	*p* Value
Intraoperative hypothermia	4.879 (3.020–7.882)	<.001	2.938 (1.310–6.589)	.009	2.562 (1.089–6.028)	.031

*Note*: Adjusted1: adjusted for age (40–90), gender, education, and MMSE. Adjusted2: adjusted for age (40–90), gender, education, MMSE, smoking history, drinking history, hypertension, diabetes, and CHD.

### 3.5 Predictive model

**FIGURE 2:**
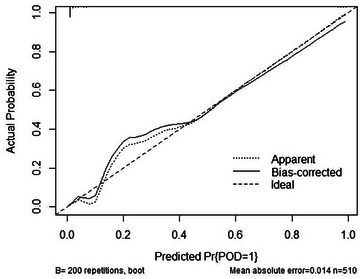
The Calibration curve. The prediction effect of the nomogram is good.

**FIGURE 3:**
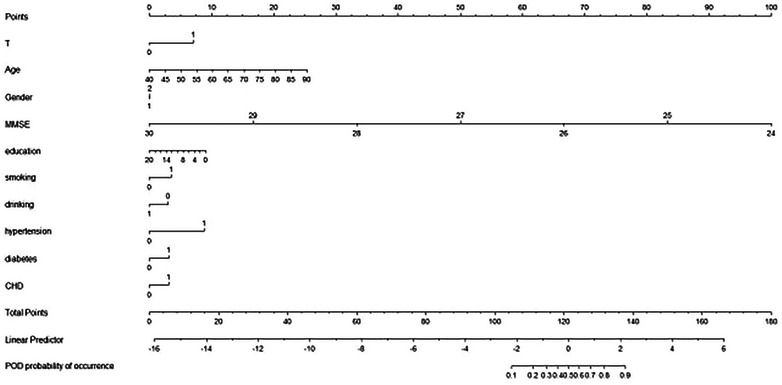
The efficacy of each predictor is shown in the nomogram. Intraoperative hypothermia is one of the risk factors for postoperative delirium (POD), and the occurrence of intraoperative hypothermia increases the risk of POD. In addition, age, gender, education, Mini‐Mental State Examination (MMSE), smoking history, drinking history, hypertension, diabetes, and cardiovascular heart disease (CHD) were all influencing factors.

**FIGURE 4:**
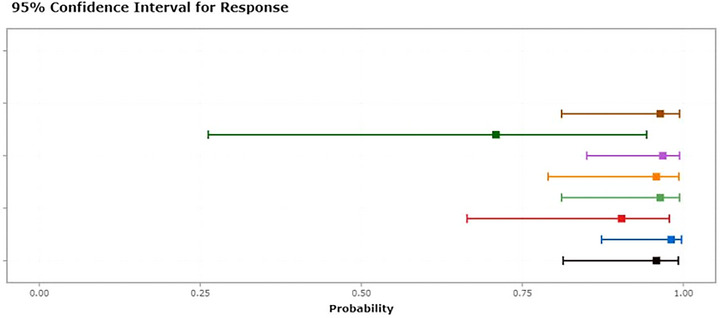
Dynamic nomogram. *Source*: Data for external validation were obtained from Qilu University School of Medicine; URL: https://thedile.shinyapps.io/dynnomapp‐1/.

The outcomes from the decision curve analysis indicate that intraoperative hypothermia exhibits a predictive effect on POD (Figure 2). The predictive efficacy of each factor is visually represented in the nomogram (Figure 3). For internal validation, we utilized data from the PNDRFAP study, revealing that intraoperative hypothermia heightens the risk of developing POD (Figure 4).

### 3.6 Causal mediation analyses

**FIGURE 5:**
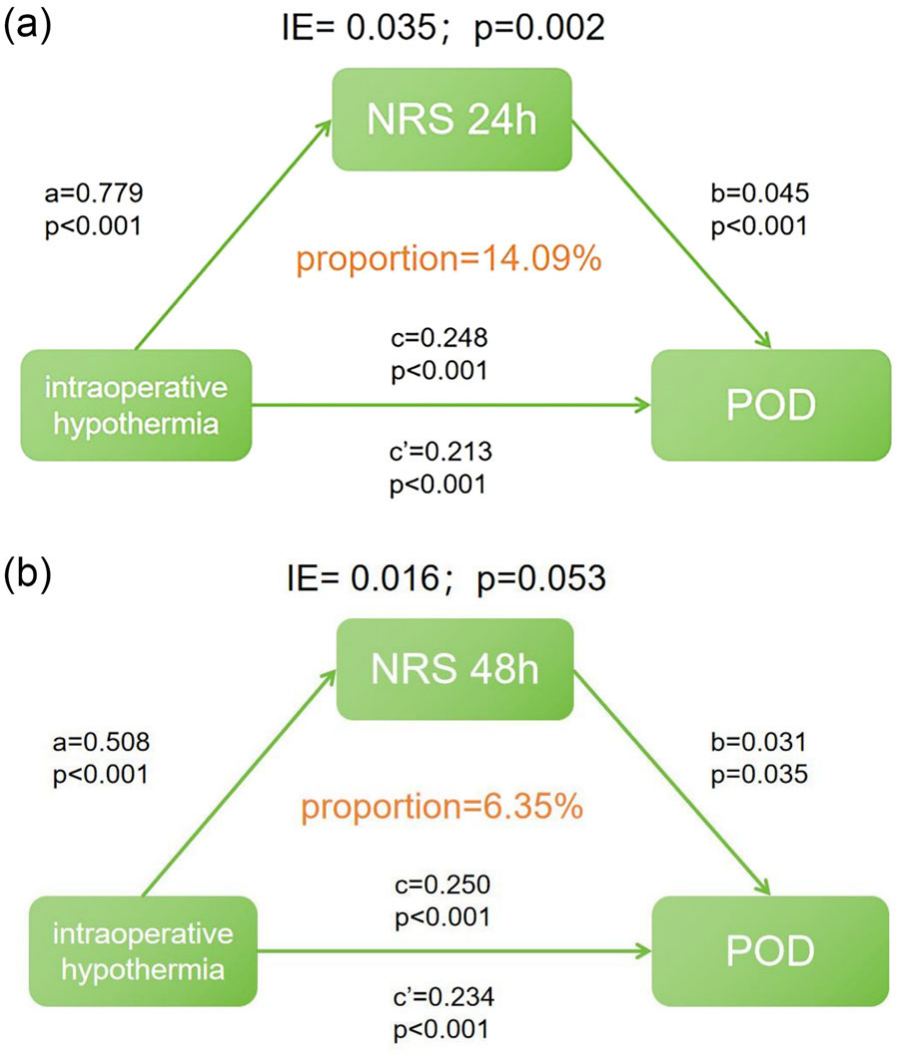
(a) The meditation analysis showed that the relationship between intraoperative hypothermia and postoperative delirium (POD) was partially meditated by NRD scores 24 h after surgery (proportion = 14.09%, *p* = .002). (b) The mediation analysis showed that the relationship between intraoperative hypothermia and POD was not mediated by numeric rating scale (NRS) scores 48 h after surgery (proportion = 6.35%, *p* = .053).

The multivariate regression analysis of the PNDRFAP study identified intraoperative hypothermia as a risk factor for POD. Consequently, we hypothesized that intraoperative hypothermia might influence the occurrence of POD through intermediary factors. To delve deeper, we investigated whether NRS scores at 24 and 48 h after surgery could mediate the impact of intraoperative hypothermia on POD. The findings indicated that the association between intraoperative hypothermia and POD was partially mediated by the NRS score 24 h after surgery, with an intermediate ratio of approximately 14.09% (*p* = .002) (Figure 5a). However, the NRS score within 48 h did not mediate this relationship (the mediation ratio was approximately 6.35%, *p* = .053) (Figure 5b).

### 3.7 ROC curve

**FIGURE 6:**
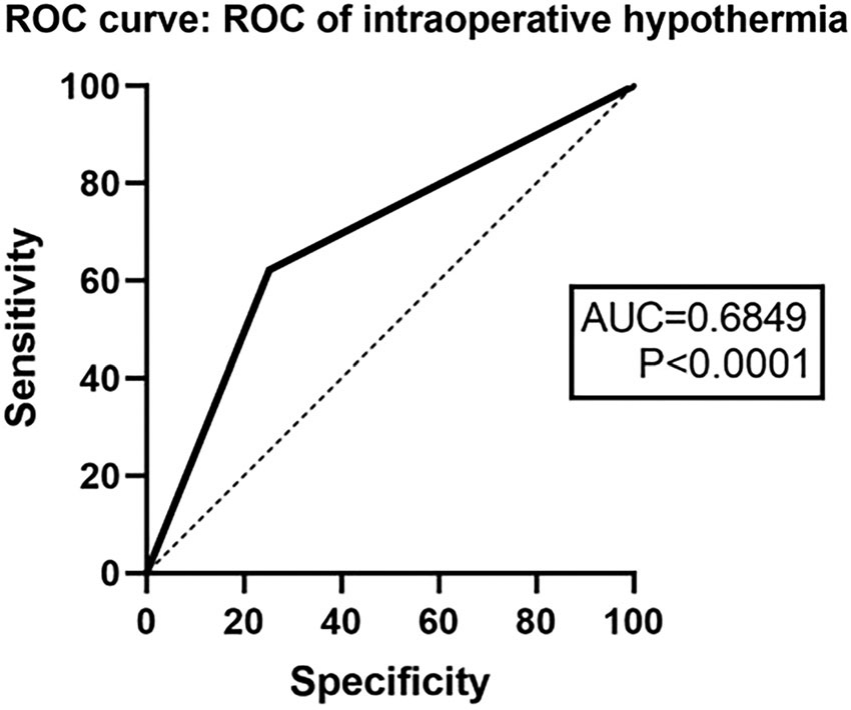
The receiver‐operating characteristic (ROC) curve showed that the intraoperative hypothermia could predict the occurrence of postoperative delirium (POD) to a certain extent in area under the curve (AUC = .6849).

The ROC curve demonstrated that the occurrence of hypothermia during the operation (AUC = .6849) could predict the occurrence of POD to a certain extent (Figure 6).

## 4 DISCUSSION

In this investigation involving 510 patients under general anesthesia, our analysis revealed that intraoperative hypothermia emerged as a risk factor for POD. Notably, patients experiencing intraoperative hypothermia exhibited diminished overall cognitive function post‐surgery. The underlying mechanism of this association appears to be linked to cerebral blood perfusion, energy metabolism, stress response, and protein deposition in cerebrospinal fluid (CSF), as suggested by previous studies (Daulatzai, 2010; Gratuze et al., 2017; Wagner et al., 2021). These findings underscore the pivotal role of intraoperative body temperature in the context of postoperative cognitive decline.

POD is a prevalent complication affecting the central nervous system in individuals following surgery. It is an acute attention and cognitive disorder with common symptoms, including confusion, attention decline, impaired orientation, cognitive decline, personality and mood changes, and even delusions and hallucinations (Aldecoa et al., 2017; Rudolph & Marcantonio, 2011; Wilson et al., 2020). It typically manifests 1–7 days after surgery (or before discharge), with a higher incidence observed within the initial 1–3 days postoperatively. Research indicates that the pathogenesis of POD encompasses various factors: (1) cholinergic theory, (2) abnormal energy metabolism, (3) inflammatory response theory, (4) oxidative stress response theory, (5) exogenous toxins and free radical damage, and (6) ischemic hypoxic brain injury. Due to the increase in life expectancy, surgical techniques are gradually improving, and more and more elderly people have the opportunity to undergo surgical treatment (Aldecoa et al., 2017).

POD is a syndrome resulting from the interplay of patient demographic factors, underlying medical conditions, anesthesia, and surgical factors. Research has highlighted a heightened incidence of POD in older patients with preexisting psychiatric disorders, particularly following emergency or complex surgical procedures.

Body temperature plays a crucial role in sustaining the daily metabolism and normal physiological activities of the body. Perioperative hypothermia is one of the common complications in the perioperative period, which refers to the decrease of the patient's core temperature due to various reasons. The factors of intraoperative hypothermia include environmental and psychological factors, which lead to hypothermia before anesthesia induction (Lim & Lee, 2020; Wetz et al., 2016; Whittington et al., 2010). A wide range of preoperative disinfection and prolonged exposure of intraoperative organ tissues resulted in intraoperative hypothermia (Radauceanu et al., 2009). The effect of anesthetic leads to the imbalance of heat production and heat dissipation (Kurz et al., 1995; Matsukawa et al., 1995). Anesthetic drugs have the functions of dilating blood vessels and inhibiting temperature regulation. In particular, general anesthesia causes nerve block in patients, and the body's compensation for environmental temperature is reduced, resulting in a temperature drop. According to research reports, about 50% of surgical patients have a postoperative body temperature lower than 36°C, and 33% have a body temperature lower than 35°C. Hypothermia has been identified to impact cognitive function in patients, and contemporary research indicates its association as one of the risk factors for Alzheimer's disease (Whittington et al., 2010). Given the nature of gastrointestinal surgery, including the trauma involved, the extent and duration of the operation, and the effects of anesthesia, intraoperative hypothermia is prone to occurrence. This increased incidence contributes to postoperative complications, ultimately influencing the prognosis of patients. Intraoperative hypothermia will affect the coagulation function of patients, increase the probability of bleeding, affect the immune function, increase the probability of tumor recurrence, increase the risk of postoperative infection, and lead to delayed postoperative recovery (Brown et al., 2017; Riley & Andrzejowski, 2018).

In hot or cold environments, the elderly have reduced basal metabolism, reduced chill intensity, reduced heat generated by reduced muscle mass, reduced body fat, reduced sensitivity to cold and temperature changes, and abnormal vasoconstriction response to cold. The ability to regulate core temperature is not as good as that of young people, and many elderly people have lower‐than‐normal body temperatures (Sessler, 2016). Therefore, recognizing and addressing intraoperative hypothermia has the potential to enhance patient outcomes, decrease the incidence of POD, improve overall patient well‐being, and alleviate the financial burden on patients (Maldonado, 2017). It is essential to acknowledge that additional research is required to elucidate the prevention of postoperative cognitive decline (Aldecoa et al., 2023; Hughes et al., 2020) and the intricate relationship between intraoperative hypothermia and POD. Further investigations are warranted to validate these findings and explore effective therapeutic interventions for managing POD (Dong et al., 2019).

Various mainstream theories have been proposed to elucidate the potential mechanism linking hypothermia to postoperative cognitive decline. One prominent theory suggests that intraoperative hypothermia leads to an upsurge in tau phosphorylation in the CSF (Wang & Mandelkow, 2016). CSF biomarkers, including amyloid β 42, total tau, and phospho‐tau, have been recognized as indicators of the POD process. The tau protein is involved in various cellular functions, including the formation of microtubules, maintenance of axon stability, development of neurons, and maintenance of neuronal polarity. Animal experiments have shown that low body temperature is associated with tau hyperphosphorylation, and this effect can be reversed when the animals are returned to normal body temperature. These findings demonstrate that anesthesia‐induced hypothermia contributes to tau hyperphosphorylation (Planel et al., 2007). In addition, the tau protein is mainly dephosphorylated by protein phosphatase type (PP‐2A). Low temperature inhibits the activity of this enzyme, resulting in excessive phosphorylation of tau protein, decreased binding ability to axon microtubules, dissociation from microtubules, and phosphorylated tau protein aggregation. Formation of paired helical filaments and further formation of neurofibrillary tangles, which cause microtubule disaggregation, cytoskeleton disintegration, and nerve cell damage, and this is the main cause of cognitive decline and memory loss in patients (Doran et al., 2007; Ihara et al., 1986; Oddo et al., 2003; Whittington et al., 2010).

According to the results of our study, patients with intraoperative hypothermia were associated with large infusion volume, intraoperative blood transfusion, large blood loss, large urine volume, intraoperative hypotension, and long anesthesia operation time. In addition, patients with intraoperative hypothermia were more likely to have delayed recovery from anesthesia and adverse reactions such as nausea and vomiting, chills, and unstable blood pressure in PACU after surgery (Yi et al., 2017). What is more, patients with intraoperative hypothermia had higher postoperative NRS scores and increased hospitalization costs. As the mediation effect analysis showed, the relationship between intraoperative hypothermia and POD was partially mediated by NRS scores 24 h after surgery, whereas it was not mediated by NRS scores 48 h after surgery. Therefore, we speculated that POD caused by intraoperative hypothermia may be related to short‐term pain. The incidence of postoperative chills increased in patients with intraoperative hypothermia during recovery from general anesthesia. Chills can increase the pain of the wound, which can cause discomfort and lead to increased use of pain medication for the patient after surgery. Increased resting pain is the only type of pain that increases the incidence of POD by affecting the sleep–wake cycle and hormonal environment, which is often associated with POD (de Heer et al., 2018; Lynch et al., 1998). What is more, the muscle activity caused by shivering increases oxygen consumption, which is a great burden for patients with poor cardiopulmonary function. Hence, the management of intraoperative body temperature and the reduction of postoperative short‐term pain can effectively reduce the occurrence of POD and improve the prognosis, comfort, and satisfaction of patients.

Therefore, for surgical patients, we should routinely carry out intraoperative temperature monitoring. Intraoperative nasopharyngeal temperature monitoring is often used to continuously monitor temperature changes and provide timely treatment, which better reflects brain temperature. For insulation measures, we can use environmental temperature maintenance, infusion heating, heaters, and other measures (Andrzejowski et al., 2010; Lau et al., 2018).

However, this study explored the effect of mild intraoperative hypothermia on patients, which is different from therapeutic hypothermia on cognitive function (Agnoletti et al., 2005; Chen et al., 2020; Foudraine et al., 2021). Some studies have shown that low temperature has a brain‐protective effect, and low temperature may be beneficial to certain critically ill patients, such as patients with craniocerebral injury (Li et al., 2012). Therapeutic hypothermia includes mild hypothermia (28–35°C) and deep hypothermia (17–28°C). Low temperature will inhibit the central nervous system, thereby reducing brain metabolic rate, cerebral blood flow, cerebral oxygen consumption, lactic acid accumulation, and free radical production (Fukuda & Warner, 2007). Low temperatures could impair blood clotting, which improves microcirculation in the brain. Low temperature could also reduce cerebral perfusion pressure, reduce intracranial pressure, and alleviate brain edema.

The limitations of this study include the fact that only the clinical characteristics of patients during the operation were studied, without further confirmation of the pathological mechanism of POD and intraoperative hypothermia. Additionally, the study was conducted on hospitalized patients scheduled for surgery, so further research is needed to validate the conclusions in animal studies. Furthermore, based on this study, a randomized controlled trial could be conducted to implement active heat preservation measures on patients to further investigate the relationship between the two groups.

## 5 CONCLUSION

In summary, intraoperative hypothermia poses a risk factor for the development of POD, making patients more susceptible to POD. This study indicates that the association between intraoperative hypothermia and POD might be partially mediated by 24‐h postoperative NRS scores. Intraoperative hypothermia shows promise as a predictor for the onset of POD. Consequently, early intervention to prevent POD has the potential to enhance postoperative quality of life, shorten hospitalization duration, and alleviate the burden on both the patient's family and society.

## AUTHOR CONTRIBUTIONS

**Jiahan Wang**: Writing—original draft; conceptualization; writing—review and editing; methodology; software; data curation. **Lei Zhu**: Conceptualization; methodology; software. **Chuan Li**: Investigation; validation; conceptualization. **Yanan Lin**: Conceptualization; formal analysis; supervision. **Bin Wang**: Conceptualization; investigation; validation; funding acquisition; visualization. **Xu Lin**: Conceptualization; investigation; validation; methodology; project administration; formal analysis; supervision; resources. **Yanlin Bi**: Conceptualization; project administration; resources; funding acquisition; visualization; formal analysis; supervision.

## CONFLICT OF INTEREST STATEMENT

The authors declare that they have no conflicts of interest with respect to the research, authorship, and/or publication of this article.

## FUNDING INFORMATION

National Natural Science Foundation of China, Grant Number: No. 91849126

### PEER REVIEW

The peer review history for this paper is available at https://publons.com/publon/10.1002/brb3.3512.

## Data Availability

The datasets used and/or analyzed during the current study are available from the corresponding author on reasonable request.

## References

[brb33512-bib-0001] Agnoletti, V. , Ansaloni, L. , Catena, F. , Chattat, R. , De Cataldis, A. , Di Nino, G. , Franceschi, C. , Gagliardi, S. , Melotti, R. M. , Potalivo, A. , & Taffurelli, M. (2005). Postoperative delirium after elective and emergency surgery: Analysis and checking of risk factors. A study protocol. BMC Surgery [Electronic Resource], 5, 12. 10.1186/1471-2482-5-12 15921527 PMC1156916

[brb33512-bib-0041] Aldecoa, C. , Bettelli, G. , Bilotta, F. , Sanders, R. D. , Aceto, P. , Audisio, R. , Cherubini, A. , Cunningham, C. , Dabrowski, W. , Forookhi, A. , Gitti, N. , Immonen, K. , Kehlet, H. , Koch, S. , Kotfis, K. , Latronico, N. , MacLullich, A. M. J. , Mevorach, L. , Mueller, A. , … Spies, C. D. (2023). Update of the European Society of Anaesthesiology and Intensive Care Medicine evidence‐based and consensus‐based guideline on postoperative delirium in adult patients, Eur J Anaesthesiol, 41(2), 81–108. 10.1097/eja.0000000000001876 37599617 PMC10763721

[brb33512-bib-0002] Aldecoa, C. , Bettelli, G. , Bilotta, F. , Sanders, R. D. , Audisio, R. , Borozdina, A. , Cherubini, A. , Jones, C. , Kehlet, H. , Maclullich, A. , Radtke, F. , Riese, F. , Slooter, A. J. C. , Veyckemans, F. , Kramer, S. , Neuner, B. , Weiss, B. , & Spies, C. D. (2017). European Society of Anaesthesiology evidence‐based and consensus‐based guideline on postoperative delirium. European Journal of Anaesthesiology, 34(4), 192–214. 10.1097/EJA.0000000000000594 28187050

[brb33512-bib-0003] Andrzejowski, J. C. , Turnbull, D. , Nandakumar, A. , Gowthaman, S. , & Eapen, G. (2010). A randomised single blinded study of the administration of pre‐warmed fluid vs active fluid warming on the incidence of peri‐operative hypothermia in short surgical procedures. Anaesthesia, 65(9), 942–945. 10.1111/j.1365-2044.2010.06473.x 20649896

[brb33512-bib-0004] Blazer, D. G. , & Van Nieuwenhuizen, A. O. (2012). Evidence for the diagnostic criteria of delirium: An update. Current Opinion in Psychiatry, 25(3), 239–243. 10.1097/YCO.0b013e3283523ce8 22449764

[brb33512-bib-0005] Brown, M. J. , Curry, T. B. , Hyder, J. A. , Berbari, E. F. , Truty, M. J. , Schroeder, D. R. , Hanson, A. C. , & Kor, D. J. (2017). Intraoperative hypothermia and surgical site infections in patients with class I/clean wounds: A case‐control study. Journal of the American College of Surgeons, 224(2), 160–171. 10.1016/j.jamcollsurg.2016.10.050 27825917

[brb33512-bib-0006] Chen, H. , Jiang, H. , Chen, B. , Fan, L. , Shi, W. , Jin, Y. , Ren, X. , Lang, L. , & Zhu, F. (2020). The incidence and predictors of postoperative delirium after brain tumor resection in adults: A cross‐sectional survey. World Neurosurgery, 140, e129–e139. 10.1016/j.wneu.2020.04.195 32376378

[brb33512-bib-0038] Daulatzai, M. A. (2010). Conversion of elderly to Alzheimer's dementia: role of confluence of hypothermia and senescent stigmata‐‐the plausible pathway. J Alzheimers Dis, 21(4), 1039–1063. 10.3233/jad-2010-100267 21504131

[brb33512-bib-0007] De Heer, E. W. , Ten Have, M. , Van Marwijk, H. W. J. , Dekker, J. , De Graaf, R. , Beekman, A. T. F. , & Van Der Feltz‐Cornelis, C. M. (2018). Pain as a risk factor for common mental disorders. Results from the Netherlands Mental Health Survey and incidence study‐2: A longitudinal, population‐based study. Pain, 159(4), 712–718. 10.1097/j.pain.0000000000001133 29252911

[brb33512-bib-0042] Dong, X. , Nao, J. , Shi, J. , & Zheng, D. (2019). Predictive value of routine peripheral blood biomarkers in alzheimer's disease. Front Aging Neurosci, 11, 332. 10.3389/fnagi.2019.00332 31866854 PMC6906180

[brb33512-bib-0008] Doran, M. , Du Plessis, D. G. , Ghadiali, E. J. , Mann, D. M. A. , Pickering‐Brown, S. , & Larner, A. J. (2007). Familial early‐onset dementia with tau intron 10 + 16 mutation with clinical features similar to those of Alzheimer disease. Archives of Neurology, 64(10), 1535–1539. 10.1001/archneur.64.10.1535 17923640

[brb33512-bib-0009] Evered, L. , Silbert, B. , Knopman, D. S. , Scott, D. A. , Dekosky, S. T. , Rasmussen, L. S. , Oh, E. S. , Crosby, G. , Berger, M. , Eckenhoff, R. G. , Evered, L. , Eckenhoff, R. G. , Ames, D. , Bekker, A. , Berger, M. , Blacker, D. , Browndyke, J. , Crosby, G. , Deiner, S. G. , … Xie, Z. (2018). Recommendations for the nomenclature of cognitive change associated with anaesthesia and surgery‐2018. British Journal of Anaesthesia, 121(5), 1005–1012. 10.1016/j.bja.2017.11.087 30325748

[brb33512-bib-0010] Forbes, S. S. , Eskicioglu, C. , Nathens, A. B. , Fenech, D. S. , Laflamme, C. , Mclean, R. F. , & Mcleod, R. S. (2009). Evidence‐based guidelines for prevention of perioperative hypothermia. Journal of the American College of Surgeons, 209(4), 492–503e1. 10.1016/j.jamcollsurg.2009.07.002 19801323

[brb33512-bib-0011] Foudraine, N. A. , Algargoush, A. , Van Osch, F. H. , & Bos, A. T. (2021). A multimodal sevoflurane‐based sedation regimen in combination with targeted temperature management in post‐cardiac arrest patients reduces the incidence of delirium: An observational propensity score‐matched study. Resuscitation, 159, 158–164. 10.1016/j.resuscitation.2020.10.042 33189803

[brb33512-bib-0012] Frank, S. M. (1997). Perioperative maintenance of normothermia reduces the incidence of morbid cardiac events. A randomized clinical trial. JAMA, 277(14), 1127–1134. 10.1001/jama.1997.03540380041029 9087467

[brb33512-bib-0013] Fukuda, S. , & Warner, D. S. (2007). Cerebral protection. British Journal of Anaesthesia, 99(1), 10–17. 10.1093/bja/aem140 17573393

[brb33512-bib-0039] Gratuze, M. , El Khoury, N. B. , Turgeon, A. , Julien, C. , Marcouiller, F. , Morin, F. , Whittington, R. A. , Marette, A. , Calon, F. , & Planel, E. (2017). Tau hyperphosphorylation in the brain of ob/ob mice is due to hypothermia: Importance of thermoregulation in linking diabetes and Alzheimer's disease. Neurobiol Dis, 98, 1–8. 10.1016/j.nbd.2016.10.004 27793638

[brb33512-bib-0043] Hughes, C. G. , Boncyk, C. S. , Culley, D. J. , Fleisher, L. A. , Leung, J. M. , McDonagh, D. L. , Gan, T. J. , McEvoy, M. D. , & Miller, T. E. (2020). American society for enhanced recovery and perioperative quality initiative joint consensus statement on postoperative delirium prevention. Anesth Analg, 130(6), 1572–1590. 10.1213/ane.0000000000004641 32022748 PMC7379173

[brb33512-bib-0014] Ihara, Y. , Nukina, N. , Miura, R. , & Ogawara, M. (1986). Phosphorylated tau protein is integrated into paired helical filaments in Alzheimer's disease. Journal of Biochemistry, 99(6), 1807–1810. 10.1093/oxfordjournals.jbchem.a135662 2427509

[brb33512-bib-0015] Kurz, A. , Go, J. C. , Sessler, D. I. , Kaer, K. , Larson, M. D. , & Bjorksten, A. R. (1995). Alfentanil slightly increases the sweating threshold and markedly reduces the vasoconstriction and shivering thresholds. Anesthesiology, 83(2), 293–299. 10.1097/00000542-199508000-00009 7631951

[brb33512-bib-0016] Lau, A. , Lowlaavar, N. , Cooke, E. M. , West, N. , German, A. , Morse, D. J. , Görges, M. , & Merchant, R. N. (2018). Effect of preoperative warming on intraoperative hypothermia: A randomized‐controlled trial. Canadian Journal of Anaesthesia, 65(9), 1029–1040. 10.1007/s12630-018-1161-8 29872966

[brb33512-bib-0017] Leslie, K. , & Sessler, D. I. (2003). Perioperative hypothermia in the high‐risk surgical patient. Best Practice & Research Clinical Anaesthesiology, 17(4), 485–498. 10.1016/S1521-6896(03)00049-1 14661653

[brb33512-bib-0018] Li, L. R. , You, C. , & Chaudhary, B. (2012). Intraoperative mild hypothermia for postoperative neurological deficits in intracranial aneurysm patients. Cochrane Database of Systematic Reviews, 00(2), Cd008445. 10.1002/14651858.CD008445.pub2 22336843

[brb33512-bib-0019] Lim, B.‐G. , & Lee, I. L.‐O. K. (2020). Anesthetic management of geriatric patients. Korean Journal of Anesthesiology, 73(1), 8–29. 10.4097/kja.19391 31636241 PMC7000283

[brb33512-bib-0020] Lynch, E. P. , Lazor, M. A. , Gellis, J. E. , Orav, J. , Goldman, L. , & Marcantonio, E. R. (1998). The impact of postoperative pain on the development of postoperative delirium. Anesthesia and Analgesia, 86(4), 781–785. 10.1097/00000539-199804000-00019 9539601

[brb33512-bib-0044] Maldonado, J. R. (2017). Acute brain failure: Pathophysiology, diagnosis, management, and sequelae of delirium. Crit Care Clin, 33(3), 461–519. 10.1016/j.ccc.2017.03.013 28601132

[brb33512-bib-0021] Matsukawa, T. , Kurz, A. , Sessler, D. I. , Bjorksten, A. R. , Merrifield, B. , & Cheng, C. (1995). Propofol linearly reduces the vasoconstriction and shivering thresholds. Anesthesiology, 82(5), 1169–1180. 10.1097/00000542-199505000-00012 7741292

[brb33512-bib-0022] Oddo, S. , Caccamo, A. , Shepherd, J. D. , Murphy, M. P , Golde, T. E. , Kayed, R. , Metherate, R. , Mattson, M. P. , Akbari, Y. , & Laferla, F. M. (2003). Triple‐transgenic model of Alzheimer's disease with plaques and tangles: Intracellular Abeta and synaptic dysfunction. Neuron, 39(3), 409–421. 10.1016/S0896-6273(03)00434-3 12895417

[brb33512-bib-0023] Planel, E. , Richter, K. E. G. , Nolan, C. E. , Finley, J. E. , Liu, L. , Wen, Y. , Krishnamurthy, P. , Herman, M. , Wang, L. , Schachter, J. B. , Nelson, R. B. , Lau, L.‐F. , & Duff, K. E. (2007). Anesthesia leads to tau hyperphosphorylation through inhibition of phosphatase activity by hypothermia. Journal of Neuroscience, 27(12), 3090–3097. 10.1523/JNEUROSCI.4854-06.2007 17376970 PMC6672474

[brb33512-bib-0024] Radauceanu, D. S. , Dragnea, D. , & Craig, J. (2009). NICE guidelines for inadvertent peri‐operative hypothermia. Anaesthesia, 64(12), 1381–1382. 10.1111/j.1365-2044.2009.06141_18.x 20092528

[brb33512-bib-0025] Riley, C. , & Andrzejowski, J. (2018). Inadvertent perioperative hypothermia. BJA Education, 18(8), 227–233. 10.1016/j.bjae.2018.05.003 33456837 PMC7807998

[brb33512-bib-0026] Rudiger, A. , Begdeda, H. , Babic, D. , Krüger, B. , Seifert, B. , Schubert, M. , Spahn, D. R. , & Bettex, D. (2016). Intra‐operative events during cardiac surgery are risk factors for the development of delirium in the ICU. Critical Care (London, England), 20, 264. 10.1186/s13054-016-1445-8 27544077 PMC4992555

[brb33512-bib-0027] Rudolph, J. L. , & Marcantonio, E. R. (2011). Review articles: Postoperative delirium: Acute change with long‐term implications. Anesthesia and Analgesia, 112(5), 1202–1211. 10.1213/ANE.0b013e3182147f6d 21474660 PMC3090222

[brb33512-bib-0028] Sessler, D. I. (2000). Perioperative heat balance. Anesthesiology, 92(2), 578–596. 10.1097/00000542-200002000-00042 10691247

[brb33512-bib-0029] Sessler, D. I. (2016). Perioperative thermoregulation and heat balance. Lancet, 387(10038), 2655–2664. 10.1016/S0140-6736(15)00981-2 26775126

[brb33512-bib-0040] Wagner, D. , Hooper, V. , Bankieris, K. , & Johnson, A. (2021). The relationship of postoperative delirium and unplanned perioperative hypothermia in surgical patients. J Perianesth Nurs, 36(1), 41–46. 10.1016/j.jopan.2020.06.015 33067117

[brb33512-bib-0045] Wang, Y. , & Mandelkow, E. (2016). Tau in physiology and pathology. Nat Rev Neurosci, 17(1), 5–21. 10.1038/nrn.2015.1 26631930

[brb33512-bib-0030] Wetz, A. J. , Perl, T. , Brandes, I. F. , Harden, M. , Bauer, M. , & Bräuer, A. (2016). Unexpectedly high incidence of hypothermia before induction of anesthesia in elective surgical patients. Journal of Clinical Anesthesia, 34, 282–289. 10.1016/j.jclinane.2016.03.065 27687393

[brb33512-bib-0031] Whittington, R. A. , Papon, M.‐A. , Chouinard‐Decorte, F. , & Planel, E. (2010). Hypothermia and Alzheimer's disease neuropathogenic pathways. Current Alzheimer Research, 7(8), 717–725. 10.2174/156720510793611646 20678067

[brb33512-bib-0032] Wilson, J. E. , Mart, M. F. , Cunningham, C. , Shehabi, Y. , Girard, T. D. , Maclullich, A. M. J. , Slooter, A. J. C. , & Ely, E. W. (2020). Delirium. Nature Reviews Disease Primers, 6(1), 90. 10.1038/s41572-020-00223-4 PMC9012267 33184265

[brb33512-bib-0033] Witlox, J. , Eurelings, L. S. M. , De Jonghe, J. F. M. , Kalisvaart, K. J. , Eikelenboom, P. , & Van Gool, W. A. (2010). Delirium in elderly patients and the risk of postdischarge mortality, institutionalization, and dementia: A meta‐analysis. JAMA, 304(4), 443–451. 10.1001/jama.2010.1013 20664045

[brb33512-bib-0034] Yi, J. , Lei, Y. , Xu, S. , Si, Y. , Li, S. , Xia, Z. , Shi, Y. , Gu, X. , Yu, J. , Xu, G. , Gu, E. , Yu, Y. , Chen, Y. , Jia, H. , Wang, Y. , Wang, X. , Chai, X. , Jin, X. , Chen, J. , … Huang, Y. (2017). Intraoperative hypothermia and its clinical outcomes in patients undergoing general anesthesia: National study in China. PLoS ONE, 12(6), e0177221. 10.1371/journal.pone.0177221 28594825 PMC5464536

[brb33512-bib-0035] Yi, J. , Xiang, Z. , Deng, X. , Fan, T. , Fu, R. , Geng, W. , Guo, R. , He, N. , Li, C. , Li, L. , Li, M. , Li, T. , Tian, M. , Wang, G. , Wang, L. , Wang, T. , Wu, A. , Wu, D. , Xue, X. , … Huang, Y. (2015). Incidence of inadvertent intraoperative hypothermia and its risk factors in patients undergoing general anesthesia in Beijing: A prospective regional survey. PLoS ONE, 10(9), e0136136. 10.1371/journal.pone.0136136 26360773 PMC4567074

[brb33512-bib-0036] Zheng, J. W. , Meng, B. , Li, X. Y. , Lu, B. , Wu, G. R. , & Chen, J. P. (2017). NF‐κB/P65 signaling pathway: A potential therapeutic target in postoperative cognitive dysfunction after sevoflurane anesthesia. European Review for Medical and Pharmacological Sciences, 21(2), 394–407. 28165545

